# Crystal structures of a manganese(I) and a rhenium(I) complex of a bi­pyridine ligand with a non-coordinating benzoic acid moiety

**DOI:** 10.1107/S2056989018006047

**Published:** 2018-04-27

**Authors:** Sheri Lense, Ilia A. Guzei, Jessica Andersen, Kong Choua Thao

**Affiliations:** a University of Wisconsin Oshkosh Department of Chemistry, 800 Algoma Blvd., Oshkosh, WI 54902, USA; b Department of Chemistry, University of Wisconsin-Madison, 1101 University Ave., Madison, WI, 53706, USA

**Keywords:** crystal structure, manganese complex, rhenium complex, bipyridyl ligand, benzoic acid substituent, disorder, hydrogen bonding

## Abstract

The structures of two facially coordinated Group VII metal complexes are reported, namely: *fac*-bromido­[2-(2,2′-bipyridin-6-yl)benzoic acid-κ^2^ *N*,*N*′]tricarbonyl­manganese(I) tetra­hydro­furan monosolvate, [MnBr(C_17_H_12_N_2_O_2_)(CO)_3_]·C_4_H_8_O, **I**, and *fac*-[2-(2,2′-bipyridin-6-yl)benzoic acid-κ^2^ *N*,*N*′]tricarbonyl­chlorido­rhenium(I) tetra­hydro­furan monosolvate, [ReCl(C_17_H_12_N_2_O_2_)(CO)_3_]·C_4_H_8_O, **II**. In both complexes, the metal ion is coordinated by three carbonyl ligands, a halide ion, and a 2-(2,2′-bipyridin-6-yl)benzoic acid ligand, in a distorted octa­hedral geometry. In manganese complex **I**, the tetra­hydro­furan (THF) solvent mol­ecule could not be refined due to disorder. The benzoic acid fragment is also disordered over two positions, such that the carb­oxy­lic acid group is either positioned near to the bromide ligand or to the axial carbonyl ligand. In the crystal of **I**, the complex mol­ecules are linked by a pair of C—H⋯Br hydrogen bonds, forming inversion dimers that stack up the *a*-axis direction. In the rhenium complex **II**, there is hydrogen bonding between the benzoic acid moiety and a disordered co-crystallized THF mol­ecule. In the crystal, the mol­ecules are linked by C—H⋯Cl hydrogen bonds, forming layers parallel to (100) separated by layers of THF solvent mol­ecules.

## Abstract 2

In the title Mn^I^ and Re^I^ complexes of the ligand 2-(2,2′-bipyridin-6-yl)benzoic acid, the *o*-benzoic acid substituent does not coordinate to the metal. In *fac*-[2-(2,2′-bipyridin-6-yl)benzoic acid-κ^2^ *N*,*N*′]tri­carbonyl­chlorido­rhenium(I) tetra­hydro­furan monosolvate, the benzoic acid fragment is positioned near the axial carbonyl ligand, whereas in *fac*-[2-(2,2′-bipyridin-6-yl)benzoic acid-κ^2^ *N*,*N*′]bromido­tri­carbonyl­manganese(I) tetra­hydro­furan monosolvate, the benzoic acid fragment is disordered, such that in the major component the benzoic fragment is positioned near the bromide ligand and in the minor fragment near the axial carbonyl ligand.

## Chemical context   

Crystal structures of *fac*-[*M*(2,2′-bipyrid­yl)(CO)_3_ *X*]^*n*+^ (*M* = Mn^I^ or Re^I^, *X* = monoanionic ligand, *n* = 0 or *X* = neutral ligand, *n* = 1) complexes have been reported for complexes bearing many different bipyridyl derivatives. Among the numerous examples are structures reported by Chen *et al.* (2005 ▸), Gerlits & Coppens (2001 ▸), and Horn *et al.* (1987 ▸). Complexes of the type *fac*-[Re(2,2′-bipyrid­yl)(CO)_3_ *X*]^*n*+^ and *fac*-[Mn(2,2′-bipyrid­yl)(CO)_3_ *X*]^*n*+^, are of particular inter­est as selective catalysts for the reduction of CO_2_ to CO (Bourrez *et al.*, 2011 ▸; Hawecker *et al.*, 1986 ▸; Smieja *et al.*, 2013 ▸; Sampson *et al.*, 2014 ▸; Machan *et al.*, 2014 ▸; Smieja & Kubiak, 2010 ▸). The addition of weak Brønsted acids such as water or methanol are necessary for the catalytic turnover of Mn complexes (Smieja *et al.*, 2013 ▸) and they also significantly increase the catalytic rate of Re complexes (Smieja *et al.*, 2012 ▸). Moreover, the use of bipyridyl ligands in these complexes containing phenolic functional groups positioned near the CO_2_ binding site, which can act as intra­molecular proton donors, have been shown to enhance catalytic performance. *fac*-Tri­carbonyl­bromido[2-(2,2′-bipyridin-6-yl-κ^2^ *N*,*N*′)phenol]manganese(I) showed enhanced catalytic activity for the reduction of CO_2_ to CO compared to *fac*-tri­carbonyl­bromido­(2,2′-bi­pyridine)­manganese(I) (Agarwal *et al.*, 2015 ▸). *fac*-Tri­carbonyl­bromido[2-(4-phenyl-2,2′-bipyridin-6-yl-κ^2^ *N*,*N*′)benzene-1,3-diol]man­ganese(I) was found to electrocatalytically reduce CO_2_ to a mixture of CO and formic acid in the absence of external Brønsted acids (Franco *et al.*, 2014 ▸). In the presence of external Brønsted acids, selectivity for formate *versus* CO was found to depend on acid strength (Franco *et al.*, 2017 ▸).
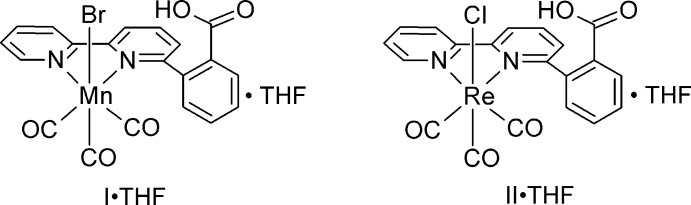


Herein, we report on the syntheses and structural characterizations of two new complexes of the type *fac*-[*M*(2,2′-bipyrid­yl)(CO)_3_ *X*]^*n*+^, *viz. fac*-bromido­[2-(2,2′-bipyridin-6-yl)benzoic acid-κ^2^ *N*,*N*′]tri­carbonyl­manganese(I) tetra­hydro­furan monosolvate, **I**, and *fac*-[2-(2,2′-bipyridin-6-yl)benzoic acid-κ^2^ *N*,*N*′]tri­carbonyl­chlorido­rhenium(I) tetra­hydro­furan monosolvate, **II**, in which the bipyridyl ligand contains a different type of intra­molecular proton donor positioned near the CO_2_ binding site. These complexes are the first reported examples of a bipyridyl ligand containing a 2-(2,2′-bipyridin-6-yl)benzoic acid backbone in which the benzoic acid moiety remains protonated and does not coordinate to the metal.

## Structural commentary   

**Figure 1:**
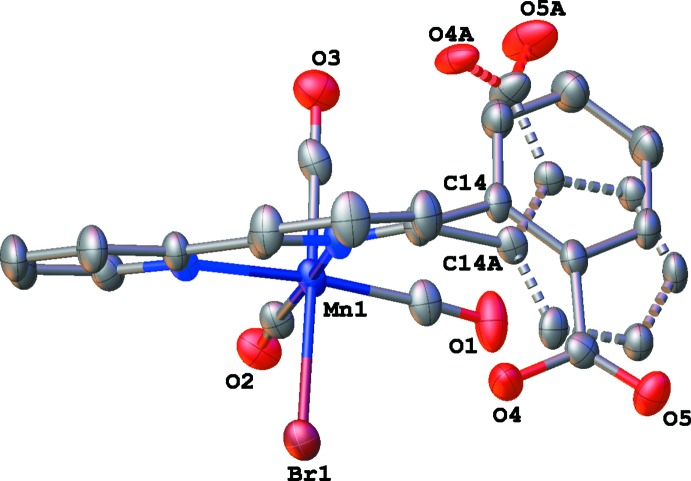
The mol­ecular structure of compound **I**, with partial atom labeling and 50% probability displacement ellipsoids. Both disorder components of the benzoic acid group are shown (the *minor* one with dashed lines), and H atoms have been omitted for clarity.

The mol­ecular structures of compounds **I** and **II** are illustrated in Figs. 1 ▸ and 2 ▸, respectively. Both compounds crystallize as tetra­hydro­furan (THF) monosolvates, THF having been used for the recrystallization of both compounds. The metal atoms exhibit distorted octa­hedral geometries and contain primary coordination spheres similar to those of other *fac*-[Re(α-di­imine)(CO)_3_Cl] and *fac*-[Mn(α-di­imine)(CO)_3_Br] complexes; including *fac*-tri­carbonyl­chlorido­(4,4′-dihy­droxy-2,2′-bi­pyri­dine)­rhenium(I) (**III**; Manbeck *et al.*, 2015 ▸), *fac*-tri­carbonyl­iodido­(2,2′-bi­pyridine)­manganese(I) (**IV**; Stor *et al.*, 1995 ▸), and *fac*-tri­carbonyl­bromido­[2-(2,2′-bipyridin-6-yl-κ^2^ *N*,*N*′)phenol]manganese(I) (**V**; Agarwal *et al.*, 2015 ▸). The metal–ligand bond distances are similar to those previously reported for complexes of this type, for *e.g.*, in **I** the Mn—N bond distances are 2.029 (2) and 2.082 (2) Å, while in **V** the Mn—N bond distances are 2.0347 (8) and 2.091 (1) Å.

**Figure 2:**
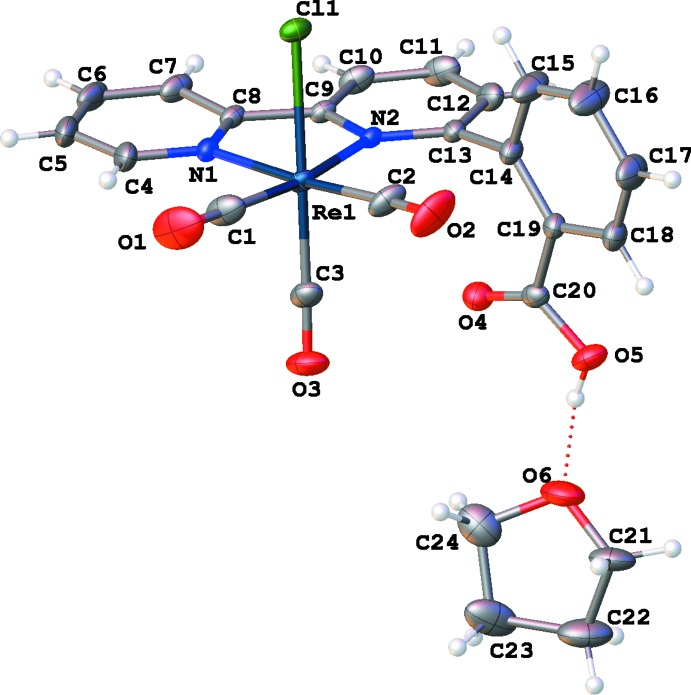
A mol­ecular structure of compound **II**, with atom labeling and 50% probability ellipsoids. The two minor solvent disorder components have been omitted for clarity

**Figure 3:**
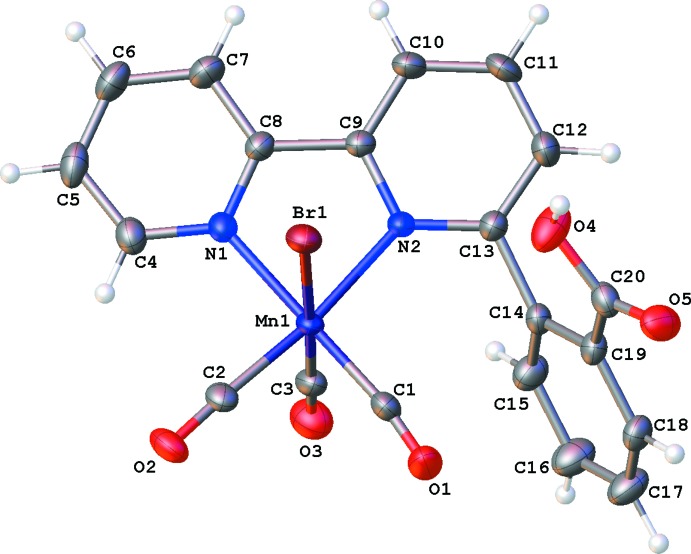
The mol­ecular structure of compound **I**, with atom labeling and showing the position of the major component of the disordered benzoic acid group. Displacement ellipsoids are drawn at the 50% probability level.

**Figure 4:**
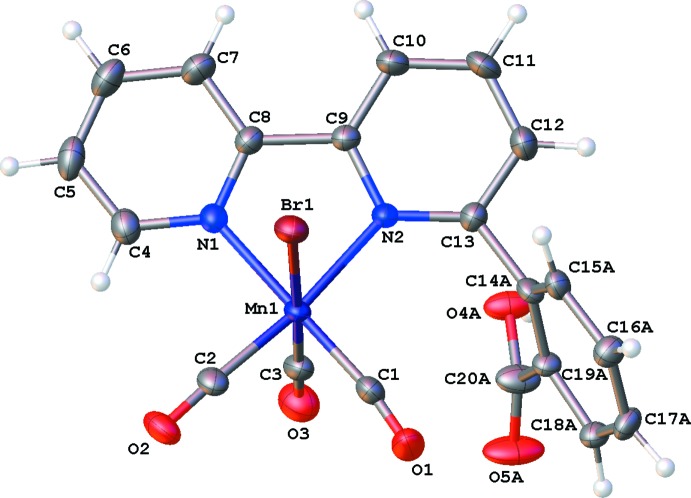
The mol­ecular structure of compound **I**, with atom labeling and showing the position of the minor component of the disordered benzoic acid group. Displacement ellipsoids are drawn at the 50% probability level.

In **I**, the benzoic acid fragment is disordered over two positions (Fig. 1 ▸). In the major component, the carb­oxy­lic acid group is positioned near the bromide ligand (see Fig. 3 ▸), whereas in the minor component the benzoic acid fragment is rotated such that the carb­oxy­lic acid group is positioned near the axial carbonyl ligand (see Fig. 4 ▸). In **II**, the benzoic acid fragment is not disordered, and the carb­oxy­lic acid group is positioned near the axial carbonyl ligand (Fig. 2 ▸).

Mol­ecules with similar motifs, in which a benzoic acid is bound to a pyridyl ring in the *ortho* position (Charris-Molina *et al.*, 2017 ▸) or to a phenyl ring in the *ortho* position (Dobson & Gerkin, 1998 ▸), have been structurally characterized. Compared to the torsion angles between the benzoic acid fragment and the pyridyl or phenyl rings in these structures, the benzoic acid fragment and the pyridyl ring in **I** and **II** are closer to being perpendicular to each other, with the N2—C13—C14—C19 torsion angle being −116.4 (3)° in the major component of **I** and 100.55 (19)° in **II**. In the minor component of **I**, the N2—C13—C14*A*—C19*A* torsion angle is 85.7 (8)°. In contrast, for the structures reported by Charris-Molina *et al.* (2017 ▸) the analogous torsion angles are 52.6 (4), −40.5 (3), −51.5 (5) and 48.8 (3)°. In the structure of biphenyl-2-carb­oxy­lic acid itself (Dobson & Gerkin, 1998 ▸), the analogous torsion angles of the four mol­ecules of the asymmetric unit are −46.7 (4), −52.3 (4), 48.2 (4) and 52.3 (4)°. Smaller absolute values of the torsion angles for **I** and **II** would result in closer contacts between the atoms of the benzoic acid fragment and the ancillary ligands around the metal, which may explain the more perpendicular torsion angles found in **I** and **II**.

## Supra­molecular features   

**Figure 5:**
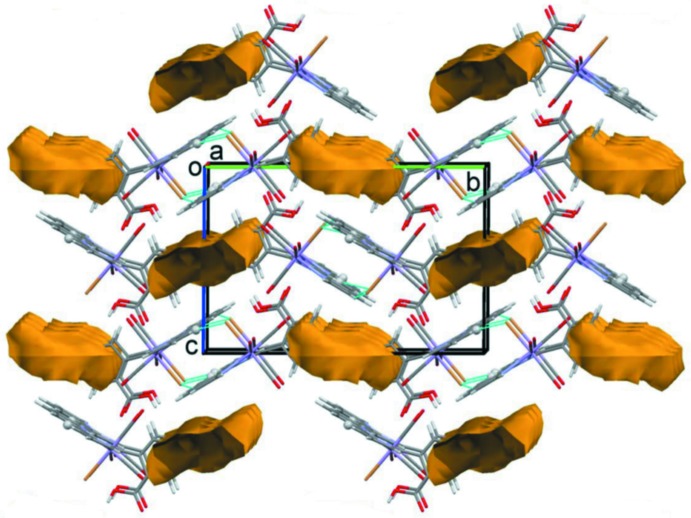
A view along the *a* axis of the crystal packing of compound **I**. The C—H⋯Br hydrogen bonds (Table 1 ▸) are shown as dashed lines and the regions occupied by the disordered THF solvent mol­ecules as yellow/brown cavities (*Mercury*; Macrae *et al.*, 2008 ▸). Only the major component of the disordered benzoic acid group is shown.

In compound **I**, the THF solvate mol­ecule is disordered over several positions and probably forms inter­molecular hydrogen bonds. In the crystal, complex mol­ecules are linked by pairs of C—H⋯Br hydrogen bonds, forming inversion dimers (Table 1 ▸). A view of the crystal packing is given in Fig. 5 ▸ and shows the voids occupied by the disordered THF solvent mol­ecules.

**Figure 6:**
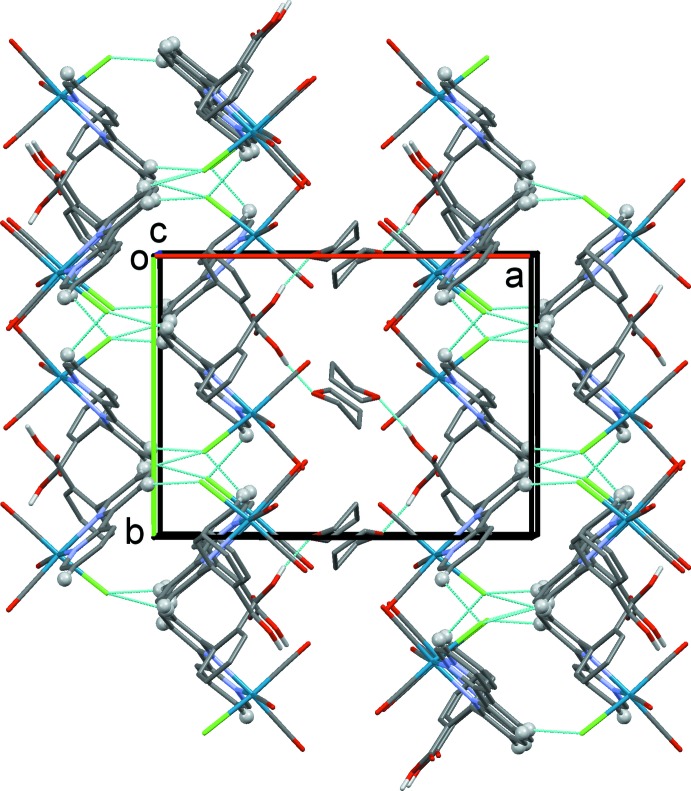
A view along the *c* axis of the crystal packing of compound **II**. The O—H⋯O and C—H⋯Cl hydrogen bonds are shown as dashed lines (Table 2 ▸). Only the major component of the disordered THF mol­ecule is shown and H atoms not involved in these inter­actions have been omitted for clarity.

In compound **II**, there is hydrogen bonding between the benzoic acid group and the oxygen atom of the disordered THF mol­ecule (Table 2 ▸). In the crystal, the complex mol­ecules are linked by C—H⋯Cl hydrogen bonds, forming layers lying parallel to the *bc* plane (Table 2 ▸ and Fig. 6 ▸), which are separated by layers of THF solvent mol­ecules.

## Database survey   

A search of the Cambridge Structural Database (Version 5.39, last update February 2018; Groom *et al.*, 2016 ▸) for complexes containing a polypyridine ligand with an *o*-benzoate substituent in the 6-position of the polypyridine moiety gave two hits, *viz*. [2-(2,2′:4′,2′′-terpyridin-6′-yl-κ^2^ *N* ^1^,*N* ^1′^)benzoato-*κO*]manganese(II) trihydrate (CSD refcode MEWBAT; Liu, 2013 ▸) and {[dimeth­yl(phen­yl)sil­yl]acetato}-[*N*-(3,5-di-*t*-butyl­phen­yl)-2-{6-[3,5-di-*t*-butyl-2-({[tris­(penta­fluoro­phen­yl)-λ^5^-boran­yl]­oxy}carbon­yl)phen­yl]pyridin-2-yl}quinolin-8-amine]­scandium toluene solvate (RIPLOT; LeBlanc *et al.*, 2014 ▸). Unlike in **I** and **II**, the benzoate substituent in these complexes is deprotonated and coordinates to the metal forming a seven-membered chelate ring. The reaction conditions and the low oxidation number of the metals in **I** and **II** would be expected to disfavor deprotonation of the benzoic acid substituent and its coordination to the metal.

## Electrochemistry   

In order to determine whether **I** and **II** could act as pre-catalysts for the reduction of CO_2_, cyclic voltammetry experiments were performed. These studies were conducted in aceto­nitrile containing 1 m*M* **I** or **II** and 0.1 *M* tetra­butyl­ammonium hexa­fluoro­phosphate using a glassy carbon working electrode, a platinum wire auxiliary electrode, and an Ag/Ag^+^ non-aqueous reference electrode. Ferrocene was used as an inter­nal standard. In order to determine whether a catalytic current enhancement was observed in the presence of the substrate, the current response was measured under an inert gas atmosphere, after bubbling CO_2_ through the solution, and after bubbling CO_2_ through the solution in the presence of an external Brønsted acid (5% water by volume). In the presence of CO_2_ and water, similar complexes have shown a catalytic current enhancement at the potential at which the complexes undergo a second one-electron reduction (Bourrez *et al.*, 2011 ▸; Agarwal *et al.*, 2015 ▸; Smieja & Kubiak, 2010 ▸). Inter­estingly, neither complex presented here showed electrocatalytic activity for the reduction of CO_2_ at or near this potential, even in the presence of an external Brønsted acid (5% water by volume). In order to probe whether catalysis was inhibited specifically by the intra­molecular nature of the benzoic acid substituent, the cyclic voltammetry of *fac*-[Mn(2,2′-bipyrid­yl)(CO)_3_Br] was performed in the presence of CO_2_, 5% water, and up to 50 molar equivalents of benzoic acid. Even in the presence of 50 molar equivalents of benzoic acid, the current enhancement was similar to that in the presence of only CO_2_ and 5% water (Bourrez *et al.*, 2011 ▸), indicating that it is the presence of the benzoic acid substituent in an intra­molecular fashion that inhibits catalysis.

## Synthesis and crystallization   

Toluene, ethanol, and aceto­nitrile used in syntheses were degassed by sparging with N_2_. THF, hexane, and pentane were dried over mol­ecular sieves and degassed using the freeze-pump-thaw method when used for recrystallization. All other reagents and solvents were purchased commercially and used as received. Metallated complexes were manipulated and stored in the dark to minimize exposure to light.

**Synthesis of methyl 2-(2,2′-bipyridin-6-yl)benzoate:** The reagents 6-bromo-2,2′-bi­pyridine (0.500 g, 2.13 mmol) and 2-meth­oxy­carbonyl­phenyl­boronic acid, pinacol ester (0.715 g, 2.73 mmol) and the catalyst tetra­kis­(tri­phenyl­phosphine)palladium(0) (0.11 g, 0.095 mmol) were placed in a Kjedahl-shaped Schlenk flask. The flask was then evacuated and refilled with nitro­gen three times, ending with the flask under nitro­gen. Toluene (26 ml), ethanol (2.6 ml), and 2 *M* aqueous K_2_CO_3_ (2.1 ml) were added to the flask, which was then heated at 368 K under nitro­gen under stirring for 41 h. The reaction mixture was cooled to room temperature, and then saturated aqueous ammonium chloride (26 ml) and deionized water (26 ml) were added to the reaction flask. The product mixture was then extracted with di­chloro­methane three times (42 ml, 30 ml, 25 ml). The combined organic layers were dried over magnesium sulfate and then filtered. The solvent was removed under vacuum. The product was purified by column chromatography using silica gel 60 as the solid phase and diethyl ether as the eluant (*R_f_* = 0.60). (yield 0.395 g, 63.9%) ^1^H NMR (270 MHz, CDCl_3_) (ppm): δ8.69 (*d*, 1H, *J* = 4.7 Hz), δ8.51–8.48 (*m*, 2H), δ7.95–7.84 (*m*, 2H), δ7.77 (*dd*, 1H, *J* = 7.5 Hz, *J* = 1.3 Hz), δ7.69–7.45 (*m*, 4H), δ7.34 (poorly resolved multiplet, 1H), δ3.53 (*s*, 3H). MS (ES–API): found *m*/*z* = 291.1 [*M* + H]^+^; {C_18_H_15_N_2_O_2_^+^} requires 291.1.

**Synthesis of 2-(2,2′-bipyridin-6-yl)benzoic acid:** Water (7.7 ml) containing 0.77 g (19 mmol) of dissolved sodium hydroxide was added to methyl 2-(2,2′-bipyridin-6-yl)benzoate (0.175 g, 0.60 mmol). The reaction mixture was refluxed for 3 h under stirring. The reaction was then cooled to room temperature. Aqueous hydro­chloric acid (2 *N*) was added dropwise until the pH was approximately 4. The white precipitate that appeared was collected on a Büchner funnel by vacuum filtration. The precipitate was washed with 4 ml deionized water and then dried *in vacuo* (yield 0.167g, 100%). ^1^H NMR (270 MHz, D_2_O containing 1*M* NaOH) (ppm): δ8.84 (*d*, 1H, *J* = 4.6 Hz), δ8.46 (*d*, 1H, *J* = 8.1 Hz), δ8.21–8.29 (*m*, 3H), δ7.88–9.95 (*m*, 2H), δ7.71–7.81 (*m*, 4H).

**Synthesis of compound I:** Bromo­penta­carbonyl­manganese(I) (0.0525 g, 0.191 mmol) and 2-(2,2′-bipyridin-6-yl)benzoic acid (0.0500 g, 0.181 mmol) were placed in a Schlenk flask, which was then evacuated and refilled with nitro­gen three times, ending with the flask under nitro­gen. Aceto­nitrile (9.4 ml) was added to the flask, which was then covered with aluminium foil. The reaction was heated to 333 K and stirred under a nitro­gen atmosphere for 12 h. The reaction was cooled to room temperature and the solvent then removed under vacuum. The crude product was dissolved in a minimal amount of THF and recrystallized by slow diffusion of hexane into the solution. These recrystallization conditions performed on a smaller scale produced the yellow needle-like crystals used for X-ray crystallographic analysis. IR ν_CO_ (KBr pellet, cm^−1^): 2019(*s*), 1933(*s*), 1912(*s*).

**Synthesis of compound II:** Penta­carbonyl­chloro­rhenium(I) (0.0273 g, 0.0755 mmol) and 2-(2,2′-bipyridin-6-yl)benzoic acid (0.0207 g, 0.0749 mmol) were placed in a Schlenk flask, which was then evacuated and refilled with nitro­gen three times, ending with the flask under nitro­gen. Aceto­nitrile (3.5 ml) was added to the flask, which was then covered with aluminium foil. The reaction was heated to 333 K and stirred under a nitro­gen atmosphere for 12 h. The reaction was cooled to room temperature and the solvent then removed under vacuum. The crude product was dissolved in a minimal amount of THF and recrystallized by slow diffusion of pentane into the solution. These recrystallization conditions performed on a smaller scale produced the yellow block-like crystals used for X-ray crystallographic analysis. IR ν_CO_ (KBr pellet, cm^−1^): 2072(*s*), 1977(*s*), 1936(*s*).

## Refinement   

Crystal data, data collection and structure refinement details are summarized in Table 3 ▸. For both compounds the C-bound H atoms were included in idealized positions and allowed to ride on the parent atoms: C—H = 0.95–0.99 Å with *U*_iso_(H) = 1.2*U*_eq_(C). In compound **II**, the carb­oxy­lic H atom was located in a difference-Fourier map and freely refined.

In compound **I**, the benzoic acid fragment is disordered over two positions with the major component contribution of 74.8 (3)%. The disordered fragment was refined with restraints. The solvent THF mol­ecule is disordered over at least three positions. Bond-length restraints were applied to model the mol­ecules but the resulting isotropic displacement coefficients suggested they were mobile. In addition, the refinement was computationally unstable. Finally the option SQUEEZE of the program *PLATON* (Spek, 2015 ▸) was used to correct the diffraction data for diffuse scattering effects and to identify the solvate mol­ecule. *PLATON* calculated the upper limit of volume that can be occupied by the solvent to be 475 Å^3^, or 21% of the unit-cell volume. The program calculated 153 electrons in the unit cell for the diffuse species. This closely corresponds to four mol­ecules of THF (160 electrons) per unit cell, or one THF mol­ecule per Mn^I^ complex (**I**). Their formula mass and unit-cell characteristics were not taken into account during refinement. It is very likely that this solvate mol­ecule, which is disordered over several positions, could form hydrogen bonds.

In compound **II**, the THF mol­ecule is disordered over three positions in a 0.672 (3):0.202 (2):0.126 (3) ratio. The disordered mol­ecules were refined with restraints and constraints (Guzei, 2014 ▸).

## Supplementary Material

Crystal structure: contains datablock(s) I, II, Global. DOI: 10.1107/S2056989018006047/su5439sup1.cif

Structure factors: contains datablock(s) I. DOI: 10.1107/S2056989018006047/su5439Isup4.hkl

Structure factors: contains datablock(s) II. DOI: 10.1107/S2056989018006047/su5439IIsup5.hkl

CCDC references: 1838458, 1838457

Additional supporting information:  crystallographic information; 3D view; checkCIF report

## Figures and Tables

**Table 1:** Hydrogen-bond geometry (Å, °) for (I)

*D*—H⋯*A*	*D*—H	H⋯*A*	*D*⋯*A*	*D*—H⋯*A*
C10—H10⋯Br1^i^	0.95	2.87	3.685 (3)	144

Symmetry code: (i) 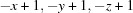.

**Table 2:** Hydrogen-bond geometry (Å, °) for (II)

*D*—H⋯*A*	*D*—H	H⋯*A*	*D*⋯*A*	*D*—H⋯*A*
O5—H5⋯O6	0.90 (3)	1.74 (4)	2.615 (4)	164 (3)
O5—H5⋯O6*A*	0.90 (3)	1.68 (4)	2.516 (15)	154 (3)
O5—H5⋯O6*B*	0.90 (3)	1.83 (4)	2.642 (9)	150 (3)
C5—H5*A*⋯Cl1^i^	0.95	2.80	3.371 (2)	120
C10—H10⋯Cl1^ii^	0.95	2.70	3.552 (2)	149
C12—H12⋯Cl1^iii^	0.95	2.76	3.524 (2)	138

Symmetry codes: (i) 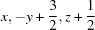; (ii) 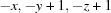; (iii) 
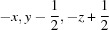
.

**Table 3:** Experimental details

	(I)	(II)
Crystal data		
Chemical formula	[MnBr(C_17_H_12_N_2_O_2_)(CO)_3_]·C_4_H_8_O	[ReCl(C_17_H_12_N_2_O_2_)(CO)_3_]·C_4_H_8_O
*M*_r_	495.17	654.07
Crystal system, space group	Monoclinic, *P*2_1_/*n*	Monoclinic, *P*2_1_/*c*
Temperature (K)	100	100
*a*, *b*, *c* (Å)	10.525 (3), 18.187 (5), 12.459 (4)	15.462 (4), 11.370 (3), 13.370 (3)
β (°)	105.928 (13)	98.023 (10)
*V* (Å^3^)	2293.2 (12)	2327.4 (10)
*Z*	4	4
Radiation type	Mo *K*α	Mo *K*α
μ (mm^−1^)	2.35	5.38
Crystal size (mm)	0.21 × 0.04 × 0.03	0.28 × 0.24 × 0.22

Data collection		
Diffractometer	Bruker SMART APEXIII area detector	Bruker SMART APEXIII area detector
Absorption correction	Multi-scan (*SADABS*; Krause *et al.*, 2015 ▸)	Analytical (*SADABS*; Krause *et al.*, 2015 ▸)
*T*_min_, *T*_max_	0.673, 0.802	0.285, 0.526
No. of measured, independent and observed [*I* > 2σ(*I*)] reflections	51901, 6992, 5393	70522, 7944, 7361
*R*_int_	0.048	0.027
(sin θ/λ)_max_ (Å^−1^)	0.716	0.742

Refinement		
*R*[*F* ^2^ > 2σ(*F* ^2^)], *wR*(*F* ^2^), *S*	0.041, 0.104, 1.01	0.016, 0.039, 1.03
No. of reflections	6992	7944
No. of parameters	346	342
No. of restraints	149	27
H-atom treatment	H-atom parameters constrained	H atoms treated by a mixture of independent and constrained refinement
Δρ_max_, Δρ_min_ (e Å^−3^)	0.59, −0.77	1.63, −1.64

Computer programs: *APEX3* and *SAINT-Plus* (Bruker, 2015 ▸), *SHELXT* (Sheldrick, 2015*a* ▸), *SHELXL* (Sheldrick, 2015*b* ▸), *OLEX2* (Dolomanov *et al.*, 2009 ▸), *Mercury* (Macrae *et al.*, 2008 ▸) and *PLATON* (Spek, 2009 ▸).

## supplementary crystallographic information

### fac-Bromido[2-(2,2'-bipyridin-6-yl)benzoic acid-κ2N,N']tricarbonylmanganese(I) tetrahydrofuran monosolvate (I) Crystal data

**Table:**

[MnBr(C_17_H_12_N_2_O_2_)(CO)_3_]·C_4_H_8_O	*F*(000) = 984
*M**_r_* = 495.17	*D*_x_ = 1.434 Mg m^−^^3^
Monoclinic, *P*2_1_/*n*	Mo *K*α radiation, λ = 0.71073 Å
*a* = 10.525 (3) Å	Cell parameters from 9894 reflections
*b* = 18.187 (5) Å	θ = 2.2–28.9°
*c* = 12.459 (4) Å	µ = 2.35 mm^−^^1^
β = 105.928 (13)°	*T* = 100 K
*V* = 2293.2 (12) Å^3^	Needle, yellow
*Z* = 4	0.21 × 0.04 × 0.03 mm

### fac-Bromido[2-(2,2'-bipyridin-6-yl)benzoic acid-κ2N,N']tricarbonylmanganese(I) tetrahydrofuran monosolvate (I) Data collection

**Table:**

Bruker SMART APEXIII area detector diffractometer	6992 independent reflections
Radiation source: microfocus sealed X-ray tube, Incoatec Iµs	5393 reflections with *I* > 2σ(*I*)
Mirror optics monochromator	*R*_int_ = 0.048
Detector resolution: 7.9 pixels mm^-1^	θ_max_ = 30.6°, θ_min_ = 2.0°
0.5° ω and 0.5° φ scans	*h* = −15→15
Absorption correction: multi-scan (SADABS; Krause *et al.*, 2015)	*k* = −26→25
*T*_min_ = 0.673, *T*_max_ = 0.802	*l* = −17→17
51901 measured reflections	

### fac-Bromido[2-(2,2'-bipyridin-6-yl)benzoic acid-κ2N,N']tricarbonylmanganese(I) tetrahydrofuran monosolvate (I) Refinement

**Table:**

Refinement on *F*^2^	Primary atom site location: dual
Least-squares matrix: full	Hydrogen site location: inferred from neighbouring sites
*R*[*F*^2^ > 2σ(*F*^2^)] = 0.041	H-atom parameters constrained
*wR*(*F*^2^) = 0.104	*w* = 1/[σ^2^(*F*_o_^2^) + (0.0415*P*)^2^ + 3.4283*P*] where *P* = (*F*_o_^2^ + 2*F*_c_^2^)/3
*S* = 1.01	(Δ/σ)_max_ = 0.001
6992 reflections	Δρ_max_ = 0.59 e Å^−^^3^
346 parameters	Δρ_min_ = −0.76 e Å^−^^3^
149 restraints	

### fac-Bromido[2-(2,2'-bipyridin-6-yl)benzoic acid-κ2N,N']tricarbonylmanganese(I) tetrahydrofuran monosolvate (I) Special details

**Table:**

Geometry. All esds (except the esd in the dihedral angle between two l.s. planes) are estimated using the full covariance matrix. The cell esds are taken into account individually in the estimation of esds in distances, angles and torsion angles; correlations between esds in cell parameters are only used when they are defined by crystal symmetry. An approximate (isotropic) treatment of cell esds is used for estimating esds involving l.s. planes.

### fac-Bromido[2-(2,2'-bipyridin-6-yl)benzoic acid-κ2N,N']tricarbonylmanganese(I) tetrahydrofuran monosolvate (I) Fractional atomic coordinates and isotropic or equivalent isotropic displacement parameters (Å^2^)

**Table:**

	*x*	*y*	*z*	*U*_iso_*/*U*_eq_	Occ. (<1)
Br1	0.81210 (3)	0.58497 (2)	0.67241 (2)	0.03094 (8)	
Mn1	0.79215 (3)	0.67459 (2)	0.51236 (3)	0.02312 (9)	
O1	0.8538 (2)	0.79491 (12)	0.6780 (2)	0.0465 (6)	
O2	1.08314 (18)	0.67387 (11)	0.57136 (18)	0.0358 (4)	
O3	0.7716 (2)	0.77396 (12)	0.32364 (19)	0.0422 (5)	
N1	0.7657 (2)	0.58451 (10)	0.41253 (18)	0.0250 (4)	
N2	0.58835 (19)	0.66090 (10)	0.47642 (18)	0.0231 (4)	
C1	0.8213 (2)	0.74942 (14)	0.6135 (2)	0.0312 (6)	
C2	0.9700 (2)	0.67300 (13)	0.5454 (2)	0.0276 (5)	
C3	0.7791 (2)	0.73615 (14)	0.3956 (3)	0.0319 (6)	
C4	0.8611 (3)	0.55066 (14)	0.3774 (2)	0.0320 (6)	
H4	0.9461	0.5727	0.3938	0.038*	
C5	0.8408 (3)	0.48547 (15)	0.3187 (2)	0.0365 (6)	
H5	0.9105	0.4628	0.2960	0.044*	
C6	0.7168 (3)	0.45389 (15)	0.2939 (3)	0.0399 (7)	
H6	0.7000	0.4089	0.2538	0.048*	
C7	0.6172 (3)	0.48852 (15)	0.3280 (3)	0.0361 (6)	
H7	0.5313	0.4676	0.3113	0.043*	
C8	0.6446 (2)	0.55402 (13)	0.3867 (2)	0.0265 (5)	
C9	0.5448 (2)	0.59597 (12)	0.4244 (2)	0.0247 (5)	
C10	0.4168 (3)	0.57149 (15)	0.4078 (3)	0.0352 (6)	
H10	0.3897	0.5259	0.3714	0.042*	
C11	0.3283 (3)	0.61409 (16)	0.4449 (3)	0.0415 (7)	
H11	0.2408	0.5973	0.4370	0.050*	
C12	0.3689 (3)	0.68058 (15)	0.4929 (3)	0.0365 (6)	
H12	0.3088	0.7114	0.5166	0.044*	
C13	0.4982 (2)	0.70278 (13)	0.5069 (2)	0.0276 (5)	
O4	0.5438 (3)	0.69265 (14)	0.7265 (2)	0.0426 (7)	0.748 (3)
H4A	0.5453	0.6705	0.7860	0.064*	0.748 (3)
O5	0.5472 (3)	0.79126 (15)	0.8336 (2)	0.0375 (7)	0.748 (3)
C14	0.5363 (3)	0.78182 (19)	0.5386 (3)	0.0255 (7)	0.748 (3)
C15	0.5461 (5)	0.8292 (2)	0.4543 (4)	0.0335 (10)	0.748 (3)
H15	0.5330	0.8108	0.3807	0.040*	0.748 (3)
C16	0.5748 (5)	0.9035 (2)	0.4753 (4)	0.0411 (9)	0.748 (3)
H16	0.5847	0.9351	0.4174	0.049*	0.748 (3)
C17	0.5889 (4)	0.9309 (2)	0.5823 (4)	0.0393 (9)	0.748 (3)
H17	0.6067	0.9816	0.5975	0.047*	0.748 (3)
C18	0.5768 (4)	0.8846 (2)	0.6650 (4)	0.0300 (9)	0.748 (3)
H18	0.5873	0.9037	0.7379	0.036*	0.748 (3)
C19	0.5495 (3)	0.80940 (17)	0.6456 (3)	0.0260 (7)	0.748 (3)
C20	0.5467 (5)	0.7646 (2)	0.7440 (4)	0.0277 (8)	0.748 (3)
O4A	0.4563 (8)	0.8099 (4)	0.3602 (6)	0.0343 (19)	0.252 (3)
H4AA	0.4104	0.8358	0.3080	0.051*	0.252 (3)
O5A	0.5901 (11)	0.9062 (5)	0.3988 (8)	0.053 (3)	0.252 (3)
C14A	0.5335 (9)	0.7683 (5)	0.5909 (9)	0.024 (2)	0.252 (3)
C15A	0.5520 (14)	0.7576 (7)	0.7034 (10)	0.027 (3)	0.252 (3)
H15A	0.5446	0.7098	0.7319	0.033*	0.252 (3)
C16A	0.5818 (10)	0.8179 (5)	0.7748 (8)	0.028 (2)	0.252 (3)
H16A	0.5914	0.8127	0.8525	0.034*	0.252 (3)
C17A	0.5970 (11)	0.8861 (5)	0.7292 (9)	0.029 (2)	0.252 (3)
H17A	0.6121	0.9277	0.7772	0.035*	0.252 (3)
C18A	0.5916 (11)	0.8967 (6)	0.6210 (9)	0.024 (2)	0.252 (3)
H18A	0.6125	0.9429	0.5947	0.029*	0.252 (3)
C19A	0.5536 (8)	0.8360 (4)	0.5484 (7)	0.0224 (18)	0.252 (3)
C20A	0.5423 (17)	0.8528 (7)	0.4324 (10)	0.034 (3)	0.252 (3)

### fac-Bromido[2-(2,2'-bipyridin-6-yl)benzoic acid-κ2N,N']tricarbonylmanganese(I) tetrahydrofuran monosolvate (I) Atomic displacement parameters (Å^2^)

**Table:**

	*U*^11^	*U*^22^	*U*^33^	*U*^12^	*U*^13^	*U*^23^
Br1	0.02879 (13)	0.02479 (12)	0.04266 (16)	−0.00564 (9)	0.01555 (11)	0.00050 (10)
Mn1	0.02055 (17)	0.01701 (15)	0.0360 (2)	−0.00424 (12)	0.01480 (15)	−0.00188 (14)
O1	0.0328 (10)	0.0387 (11)	0.0734 (16)	−0.0104 (9)	0.0234 (11)	−0.0271 (11)
O2	0.0229 (9)	0.0385 (10)	0.0490 (12)	−0.0039 (7)	0.0148 (8)	0.0071 (9)
O3	0.0390 (11)	0.0416 (11)	0.0484 (13)	−0.0009 (9)	0.0160 (10)	0.0083 (10)
N1	0.0253 (10)	0.0208 (9)	0.0327 (11)	−0.0001 (7)	0.0144 (9)	0.0007 (8)
N2	0.0218 (9)	0.0171 (8)	0.0340 (11)	−0.0034 (7)	0.0139 (8)	−0.0027 (8)
C1	0.0220 (11)	0.0286 (12)	0.0481 (16)	−0.0055 (9)	0.0183 (11)	−0.0071 (11)
C2	0.0281 (12)	0.0219 (10)	0.0378 (14)	−0.0031 (9)	0.0172 (11)	0.0031 (10)
C3	0.0239 (12)	0.0224 (11)	0.0532 (17)	−0.0046 (9)	0.0173 (12)	−0.0046 (11)
C4	0.0303 (13)	0.0305 (12)	0.0402 (15)	0.0029 (10)	0.0182 (12)	0.0009 (11)
C5	0.0420 (15)	0.0334 (13)	0.0394 (15)	0.0132 (11)	0.0200 (13)	−0.0015 (11)
C6	0.0484 (17)	0.0271 (13)	0.0469 (17)	0.0033 (12)	0.0178 (14)	−0.0119 (12)
C7	0.0363 (14)	0.0267 (12)	0.0473 (17)	−0.0057 (10)	0.0151 (13)	−0.0132 (11)
C8	0.0287 (12)	0.0197 (10)	0.0347 (13)	−0.0025 (9)	0.0150 (10)	−0.0049 (9)
C9	0.0247 (11)	0.0197 (10)	0.0330 (13)	−0.0044 (8)	0.0134 (10)	−0.0038 (9)
C10	0.0296 (13)	0.0276 (12)	0.0539 (18)	−0.0117 (10)	0.0206 (13)	−0.0121 (12)
C11	0.0260 (13)	0.0384 (14)	0.068 (2)	−0.0138 (11)	0.0261 (14)	−0.0121 (14)
C12	0.0284 (13)	0.0325 (13)	0.0559 (18)	−0.0017 (10)	0.0237 (13)	−0.0102 (12)
C13	0.0257 (11)	0.0217 (10)	0.0387 (14)	−0.0012 (9)	0.0143 (10)	−0.0060 (10)
O4	0.068 (2)	0.0295 (13)	0.0364 (15)	0.0106 (12)	0.0239 (15)	0.0043 (11)
O5	0.0448 (16)	0.0388 (14)	0.0356 (15)	−0.0106 (12)	0.0223 (12)	−0.0067 (11)
C14	0.0217 (15)	0.0238 (16)	0.0346 (19)	0.0003 (12)	0.0137 (15)	−0.0061 (14)
C15	0.041 (2)	0.026 (2)	0.036 (2)	−0.0005 (17)	0.0158 (19)	−0.0044 (16)
C16	0.056 (3)	0.0269 (17)	0.043 (2)	−0.0034 (16)	0.0183 (19)	−0.0004 (15)
C17	0.055 (3)	0.0223 (17)	0.044 (2)	−0.0020 (16)	0.0189 (19)	−0.0034 (16)
C18	0.034 (2)	0.0257 (17)	0.034 (2)	0.0016 (14)	0.016 (2)	−0.0077 (17)
C19	0.0243 (15)	0.0233 (14)	0.0327 (17)	0.0036 (11)	0.0117 (13)	−0.0038 (13)
C20	0.0226 (17)	0.0299 (18)	0.034 (2)	0.0031 (13)	0.0132 (18)	0.0016 (16)
O4A	0.047 (4)	0.030 (3)	0.021 (3)	−0.016 (3)	0.002 (3)	0.004 (3)
O5A	0.076 (7)	0.043 (4)	0.038 (5)	−0.033 (4)	0.015 (4)	0.007 (4)
C14A	0.022 (4)	0.022 (4)	0.035 (5)	−0.004 (3)	0.017 (4)	0.000 (3)
C15A	0.023 (5)	0.028 (4)	0.036 (5)	−0.003 (3)	0.017 (4)	−0.007 (4)
C16A	0.033 (5)	0.024 (4)	0.031 (4)	−0.002 (3)	0.014 (4)	−0.003 (3)
C17A	0.039 (6)	0.019 (4)	0.037 (5)	0.001 (3)	0.021 (5)	−0.006 (3)
C18A	0.027 (5)	0.017 (4)	0.031 (5)	−0.003 (3)	0.014 (4)	−0.001 (3)
C19A	0.025 (4)	0.018 (3)	0.029 (4)	−0.004 (3)	0.014 (3)	0.000 (3)
C20A	0.046 (7)	0.033 (6)	0.026 (4)	−0.017 (5)	0.015 (4)	0.001 (3)

### fac-Bromido[2-(2,2'-bipyridin-6-yl)benzoic acid-κ2N,N']tricarbonylmanganese(I) tetrahydrofuran monosolvate (I) Geometric parameters (Å, º)

**Table:**

Br1—Mn1	2.5391 (7)	C13—C14A	1.562 (10)
Mn1—N1	2.029 (2)	O4—H4A	0.8400
Mn1—N2	2.082 (2)	O4—C20	1.326 (5)
Mn1—C1	1.822 (3)	O5—C20	1.215 (5)
Mn1—C2	1.803 (3)	C14—C15	1.384 (6)
Mn1—C3	1.810 (3)	C14—C19	1.395 (5)
O1—C1	1.139 (3)	C15—H15	0.9500
O2—C2	1.145 (3)	C15—C16	1.394 (5)
O3—C3	1.116 (3)	C16—H16	0.9500
N1—C4	1.349 (3)	C16—C17	1.392 (6)
N1—C8	1.345 (3)	C17—H17	0.9500
N2—C9	1.364 (3)	C17—C18	1.364 (6)
N2—C13	1.350 (3)	C18—H18	0.9500
C4—H4	0.9500	C18—C19	1.405 (5)
C4—C5	1.378 (4)	C19—C20	1.479 (5)
C5—H5	0.9500	O4A—H4AA	0.8400
C5—C6	1.381 (4)	O4A—C20A	1.338 (13)
C6—H6	0.9500	O5A—C20A	1.220 (12)
C6—C7	1.386 (4)	C14A—C15A	1.375 (13)
C7—H7	0.9500	C14A—C19A	1.378 (10)
C7—C8	1.386 (3)	C15A—H15A	0.9500
C8—C9	1.476 (3)	C15A—C16A	1.393 (12)
C9—C10	1.379 (3)	C16A—H16A	0.9500
C10—H10	0.9500	C16A—C17A	1.391 (12)
C10—C11	1.384 (4)	C17A—H17A	0.9500
C11—H11	0.9500	C17A—C18A	1.346 (12)
C11—C12	1.365 (4)	C18A—H18A	0.9500
C12—H12	0.9500	C18A—C19A	1.414 (11)
C12—C13	1.384 (4)	C19A—C20A	1.449 (12)
C13—C14	1.515 (4)		
			
N1—Mn1—Br1	86.05 (6)	C13—C12—H12	120.3
N1—Mn1—N2	79.15 (8)	N2—C13—C12	122.9 (2)
N2—Mn1—Br1	87.16 (6)	N2—C13—C14	117.0 (2)
C1—Mn1—Br1	88.54 (9)	N2—C13—C14A	124.3 (4)
C1—Mn1—N1	174.43 (11)	C12—C13—C14	119.3 (2)
C1—Mn1—N2	101.88 (9)	C12—C13—C14A	110.6 (4)
C2—Mn1—Br1	87.32 (8)	C20—O4—H4A	109.5
C2—Mn1—N1	94.95 (10)	C15—C14—C13	117.5 (3)
C2—Mn1—N2	172.18 (9)	C15—C14—C19	119.5 (3)
C2—Mn1—C1	83.50 (11)	C19—C14—C13	122.7 (3)
C3—Mn1—Br1	178.20 (8)	C14—C15—H15	119.4
C3—Mn1—N1	92.27 (11)	C14—C15—C16	121.3 (4)
C3—Mn1—N2	93.14 (10)	C16—C15—H15	119.4
C3—Mn1—C1	93.13 (13)	C15—C16—H16	120.4
C3—Mn1—C2	92.21 (11)	C15—C16—C17	119.2 (4)
C4—N1—Mn1	125.32 (18)	C17—C16—H16	120.4
C8—N1—Mn1	116.16 (16)	C16—C17—H17	120.2
C8—N1—C4	118.3 (2)	C18—C17—C16	119.7 (4)
C9—N2—Mn1	113.44 (15)	C18—C17—H17	120.2
C13—N2—Mn1	129.34 (16)	C17—C18—H18	119.1
C13—N2—C9	116.8 (2)	C17—C18—C19	121.9 (4)
O1—C1—Mn1	172.5 (2)	C19—C18—H18	119.1
O2—C2—Mn1	176.5 (2)	C14—C19—C18	118.5 (4)
O3—C3—Mn1	179.7 (3)	C14—C19—C20	125.1 (3)
N1—C4—H4	118.5	C18—C19—C20	116.3 (4)
N1—C4—C5	122.9 (3)	O4—C20—C19	114.3 (4)
C5—C4—H4	118.5	O5—C20—O4	122.6 (4)
C4—C5—H5	120.8	O5—C20—C19	123.1 (4)
C4—C5—C6	118.5 (2)	C20A—O4A—H4AA	109.5
C6—C5—H5	120.8	C15A—C14A—C13	121.1 (8)
C5—C6—H6	120.3	C15A—C14A—C19A	121.6 (9)
C5—C6—C7	119.3 (3)	C19A—C14A—C13	117.2 (8)
C7—C6—H6	120.3	C14A—C15A—H15A	120.5
C6—C7—H7	120.4	C14A—C15A—C16A	118.9 (11)
C6—C7—C8	119.1 (3)	C16A—C15A—H15A	120.5
C8—C7—H7	120.4	C15A—C16A—H16A	120.9
N1—C8—C7	121.8 (2)	C17A—C16A—C15A	118.2 (9)
N1—C8—C9	114.9 (2)	C17A—C16A—H16A	120.9
C7—C8—C9	123.3 (2)	C16A—C17A—H17A	118.1
N2—C9—C8	115.0 (2)	C18A—C17A—C16A	123.9 (9)
N2—C9—C10	122.5 (2)	C18A—C17A—H17A	118.1
C10—C9—C8	122.5 (2)	C17A—C18A—H18A	121.4
C9—C10—H10	120.4	C17A—C18A—C19A	117.1 (10)
C9—C10—C11	119.2 (2)	C19A—C18A—H18A	121.4
C11—C10—H10	120.4	C14A—C19A—C18A	119.8 (9)
C10—C11—H11	120.5	C14A—C19A—C20A	126.3 (9)
C12—C11—C10	118.9 (2)	C18A—C19A—C20A	113.8 (8)
C12—C11—H11	120.5	O4A—C20A—C19A	113.7 (9)
C11—C12—H12	120.3	O5A—C20A—O4A	120.0 (10)
C11—C12—C13	119.5 (2)	O5A—C20A—C19A	125.6 (11)
			
Mn1—N1—C4—C5	173.4 (2)	C12—C13—C14—C19	73.9 (4)
Mn1—N1—C8—C7	−173.9 (2)	C12—C13—C14A—C15A	72.9 (10)
Mn1—N1—C8—C9	6.8 (3)	C12—C13—C14A—C19A	−111.1 (7)
Mn1—N2—C9—C8	−9.8 (3)	C13—N2—C9—C8	176.6 (2)
Mn1—N2—C9—C10	170.4 (2)	C13—N2—C9—C10	−3.2 (4)
Mn1—N2—C13—C12	−168.8 (2)	C13—C14—C15—C16	177.1 (4)
Mn1—N2—C13—C14	21.9 (4)	C13—C14—C19—C18	−176.0 (3)
Mn1—N2—C13—C14A	−7.7 (6)	C13—C14—C19—C20	8.8 (5)
N1—C4—C5—C6	0.7 (4)	C13—C14A—C15A—C16A	−179.2 (9)
N1—C8—C9—N2	2.3 (3)	C13—C14A—C19A—C18A	−177.2 (8)
N1—C8—C9—C10	−178.0 (3)	C13—C14A—C19A—C20A	1.5 (15)
N2—C9—C10—C11	0.2 (5)	C14—C15—C16—C17	−2.5 (7)
N2—C13—C14—C15	69.6 (4)	C14—C19—C20—O4	6.6 (6)
N2—C13—C14—C19	−116.4 (3)	C14—C19—C20—O5	−173.5 (4)
N2—C13—C14A—C15A	−90.2 (10)	C15—C14—C19—C18	−2.1 (5)
N2—C13—C14A—C19A	85.7 (8)	C15—C14—C19—C20	−177.3 (4)
C4—N1—C8—C7	1.6 (4)	C15—C16—C17—C18	1.2 (7)
C4—N1—C8—C9	−177.7 (2)	C16—C17—C18—C19	−0.5 (7)
C4—C5—C6—C7	0.2 (5)	C17—C18—C19—C14	0.9 (6)
C5—C6—C7—C8	−0.2 (5)	C17—C18—C19—C20	176.6 (4)
C6—C7—C8—N1	−0.7 (4)	C18—C19—C20—O4	−168.7 (4)
C6—C7—C8—C9	178.5 (3)	C18—C19—C20—O5	11.2 (6)
C7—C8—C9—N2	−177.1 (3)	C19—C14—C15—C16	2.9 (7)
C7—C8—C9—C10	2.7 (4)	C14A—C15A—C16A—C17A	−2.7 (17)
C8—N1—C4—C5	−1.7 (4)	C14A—C19A—C20A—O4A	29 (2)
C8—C9—C10—C11	−179.6 (3)	C14A—C19A—C20A—O5A	−160.9 (15)
C9—N2—C13—C12	3.6 (4)	C15A—C14A—C19A—C18A	−1.3 (15)
C9—N2—C13—C14	−165.7 (3)	C15A—C14A—C19A—C20A	177.4 (13)
C9—N2—C13—C14A	164.7 (5)	C15A—C16A—C17A—C18A	−3.7 (17)
C9—C10—C11—C12	2.6 (5)	C16A—C17A—C18A—C19A	7.3 (17)
C10—C11—C12—C13	−2.2 (5)	C17A—C18A—C19A—C14A	−4.8 (15)
C11—C12—C13—N2	−1.0 (5)	C17A—C18A—C19A—C20A	176.4 (12)
C11—C12—C13—C14	168.0 (3)	C18A—C19A—C20A—O4A	−152.6 (12)
C11—C12—C13—C14A	−164.4 (5)	C18A—C19A—C20A—O5A	18 (2)
C12—C13—C14—C15	−100.1 (4)	C19A—C14A—C15A—C16A	5.0 (17)

### fac-Bromido[2-(2,2'-bipyridin-6-yl)benzoic acid-κ2N,N']tricarbonylmanganese(I) tetrahydrofuran monosolvate (I) Hydrogen-bond geometry (Å, º)

**Table:**

*D*—H···*A*	*D*—H	H···*A*	*D*···*A*	*D*—H···*A*
C10—H10···Br1^i^	0.95	2.87	3.685 (3)	144

Symmetry code: (i) −*x*+1, −*y*+1, −*z*+1.

### fac-[2-(2,2'-Bipyridin-6-yl)benzoic acid-κ2N,N']tricarbonylchloridorhenium(I) tetrahydrofuran monosolvate (II) Crystal data

**Table:**

[ReCl(C_17_H_12_N_2_O_2_)(CO)_3_]·C_4_H_8_O	*F*(000) = 1272
*M**_r_* = 654.07	*D*_x_ = 1.867 Mg m^−^^3^
Monoclinic, *P*2_1_/*c*	Mo *K*α radiation, λ = 0.71073 Å
*a* = 15.462 (4) Å	Cell parameters from 9101 reflections
*b* = 11.370 (3) Å	θ = 2.6–31.8°
*c* = 13.370 (3) Å	µ = 5.38 mm^−^^1^
β = 98.023 (10)°	*T* = 100 K
*V* = 2327.4 (10) Å^3^	Block, yellow
*Z* = 4	0.28 × 0.24 × 0.22 mm

### fac-[2-(2,2'-Bipyridin-6-yl)benzoic acid-κ2N,N']tricarbonylchloridorhenium(I) tetrahydrofuran monosolvate (II) Data collection

**Table:**

Bruker SMART APEXIII area detector diffractometer	7944 independent reflections
Radiation source: microfocus sealed X-ray tube, Incoatec Iµs	7361 reflections with *I* > 2σ(*I*)
Mirror optics monochromator	*R*_int_ = 0.027
Detector resolution: 7.9 pixels mm^-1^	θ_max_ = 31.9°, θ_min_ = 1.3°
0.5° ω and 0.5° φ scans	*h* = −22→22
Absorption correction: analytical (SADABS; Krause *et al.*, 2015)	*k* = −16→16
*T*_min_ = 0.285, *T*_max_ = 0.526	*l* = −19→19
70522 measured reflections	

### fac-[2-(2,2'-Bipyridin-6-yl)benzoic acid-κ2N,N']tricarbonylchloridorhenium(I) tetrahydrofuran monosolvate (II) Refinement

**Table:**

Refinement on *F*^2^	Primary atom site location: dual
Least-squares matrix: full	Hydrogen site location: mixed
*R*[*F*^2^ > 2σ(*F*^2^)] = 0.016	H atoms treated by a mixture of independent and constrained refinement
*wR*(*F*^2^) = 0.039	*w* = 1/[σ^2^(*F*_o_^2^) + (0.0182*P*)^2^ + 2.0257*P*] where *P* = (*F*_o_^2^ + 2*F*_c_^2^)/3
*S* = 1.03	(Δ/σ)_max_ = 0.003
7944 reflections	Δρ_max_ = 1.63 e Å^−^^3^
342 parameters	Δρ_min_ = −1.64 e Å^−^^3^
27 restraints	

### fac-[2-(2,2'-Bipyridin-6-yl)benzoic acid-κ2N,N']tricarbonylchloridorhenium(I) tetrahydrofuran monosolvate (II) Special details

**Table:**

Geometry. All esds (except the esd in the dihedral angle between two l.s. planes) are estimated using the full covariance matrix. The cell esds are taken into account individually in the estimation of esds in distances, angles and torsion angles; correlations between esds in cell parameters are only used when they are defined by crystal symmetry. An approximate (isotropic) treatment of cell esds is used for estimating esds involving l.s. planes.

### fac-[2-(2,2'-Bipyridin-6-yl)benzoic acid-κ2N,N']tricarbonylchloridorhenium(I) tetrahydrofuran monosolvate (II) Fractional atomic coordinates and isotropic or equivalent isotropic displacement parameters (Å^2^)

**Table:**

	*x*	*y*	*z*	*U*_iso_*/*U*_eq_	Occ. (<1)
Re1	0.25443 (2)	0.56361 (2)	0.41243 (2)	0.01643 (2)	
Cl1	0.12745 (2)	0.69931 (3)	0.37941 (3)	0.02075 (7)	
O1	0.38542 (11)	0.75829 (16)	0.48236 (17)	0.0571 (5)	
O2	0.31136 (12)	0.60610 (14)	0.20431 (13)	0.0469 (4)	
O3	0.40094 (9)	0.38300 (14)	0.46361 (13)	0.0395 (3)	
O4	0.25774 (8)	0.21703 (11)	0.31781 (9)	0.0263 (2)	
O5	0.31281 (9)	0.16759 (12)	0.17689 (11)	0.0295 (3)	
H5	0.339 (2)	0.111 (3)	0.218 (2)	0.064 (9)*	
N1	0.20926 (9)	0.54664 (12)	0.55735 (10)	0.0189 (2)	
N2	0.15082 (8)	0.42971 (10)	0.38837 (9)	0.0141 (2)	
C1	0.33623 (12)	0.68554 (17)	0.45436 (17)	0.0336 (4)	
C2	0.28734 (13)	0.58568 (16)	0.28011 (16)	0.0295 (4)	
C3	0.34552 (11)	0.44986 (15)	0.44347 (15)	0.0260 (3)	
C4	0.24129 (12)	0.60914 (17)	0.63982 (14)	0.0298 (4)	
H4	0.2891	0.6606	0.6359	0.036*	
C5	0.20740 (15)	0.6013 (2)	0.72977 (15)	0.0386 (5)	
H5A	0.2321	0.6456	0.7870	0.046*	
C6	0.13710 (14)	0.5282 (2)	0.73522 (13)	0.0365 (5)	
H6	0.1123	0.5219	0.7961	0.044*	
C7	0.10326 (12)	0.46437 (17)	0.65100 (13)	0.0271 (3)	
H7	0.0542	0.4145	0.6530	0.033*	
C8	0.14155 (10)	0.47363 (13)	0.56311 (11)	0.0178 (3)	
C9	0.11021 (9)	0.40700 (13)	0.47062 (11)	0.0163 (2)	
C10	0.04153 (11)	0.32791 (14)	0.46733 (14)	0.0256 (3)	
H10	0.0159	0.3116	0.5264	0.031*	
C11	0.01085 (12)	0.27318 (16)	0.37729 (17)	0.0313 (4)	
H11	−0.0374	0.2207	0.3729	0.038*	
C12	0.05092 (11)	0.29564 (15)	0.29444 (14)	0.0272 (3)	
H12	0.0306	0.2587	0.2318	0.033*	
C13	0.12151 (10)	0.37260 (13)	0.30166 (11)	0.0185 (3)	
C14	0.15673 (11)	0.39961 (15)	0.20576 (12)	0.0236 (3)	
C15	0.11602 (16)	0.4894 (2)	0.14585 (15)	0.0413 (5)	
H15	0.0723	0.5356	0.1704	0.050*	
C16	0.13830 (19)	0.5124 (2)	0.05091 (16)	0.0514 (7)	
H16	0.1110	0.5752	0.0116	0.062*	
C17	0.20068 (16)	0.4436 (2)	0.01340 (14)	0.0395 (5)	
H17	0.2160	0.4589	−0.0518	0.047*	
C18	0.24041 (12)	0.35285 (17)	0.07105 (12)	0.0274 (3)	
H18	0.2825	0.3052	0.0448	0.033*	
C19	0.21945 (10)	0.33025 (14)	0.16769 (12)	0.0206 (3)	
C20	0.26432 (10)	0.23343 (14)	0.23004 (13)	0.0204 (3)	
O6	0.4105 (3)	0.0039 (5)	0.2725 (3)	0.0444 (9)	0.672 (3)
C21	0.4761 (3)	−0.0296 (4)	0.2093 (3)	0.0412 (10)	0.672 (3)
H21A	0.5111	0.0390	0.1931	0.049*	0.672 (3)
H21B	0.4492	−0.0674	0.1458	0.049*	0.672 (3)
C22	0.5303 (3)	−0.1156 (4)	0.2774 (4)	0.0535 (11)	0.672 (3)
H22A	0.5900	−0.1222	0.2591	0.064*	0.672 (3)
H22B	0.5029	−0.1945	0.2737	0.064*	0.672 (3)
C23	0.5321 (2)	−0.0610 (4)	0.3845 (3)	0.0523 (11)	0.672 (3)
H23A	0.5304	−0.1234	0.4358	0.063*	0.672 (3)
H23B	0.5856	−0.0134	0.4028	0.063*	0.672 (3)
C24	0.4518 (3)	0.0153 (4)	0.3778 (3)	0.0467 (9)	0.672 (3)
H24A	0.4122	−0.0126	0.4249	0.056*	0.672 (3)
H24B	0.4678	0.0981	0.3940	0.056*	0.672 (3)
O6A	0.4080 (11)	−0.0019 (17)	0.2447 (7)	0.0339 (13)	0.202 (3)
C24A	0.4408 (8)	−0.0269 (14)	0.3488 (8)	0.0339 (13)	0.202 (3)
H24C	0.3953	−0.0664	0.3821	0.041*	0.202 (3)
H24D	0.4579	0.0469	0.3857	0.041*	0.202 (3)
C23A	0.5209 (7)	−0.1082 (11)	0.3488 (8)	0.0339 (13)	0.202 (3)
H23C	0.5763	−0.0639	0.3632	0.041*	0.202 (3)
H23D	0.5207	−0.1731	0.3981	0.041*	0.202 (3)
C22A	0.5060 (8)	−0.1537 (11)	0.2397 (8)	0.0339 (13)	0.202 (3)
H22C	0.5610	−0.1829	0.2184	0.041*	0.202 (3)
H22D	0.4617	−0.2171	0.2311	0.041*	0.202 (3)
C21A	0.4733 (14)	−0.0426 (17)	0.1815 (10)	0.0339 (13)	0.202 (3)
H21C	0.5206	0.0156	0.1796	0.041*	0.202 (3)
H21D	0.4461	−0.0610	0.1117	0.041*	0.202 (3)
O6B	0.4356 (8)	0.0219 (9)	0.2593 (10)	0.0298 (18)*	0.126 (3)
C21B	0.4661 (11)	−0.0418 (14)	0.1775 (7)	0.0298 (18)*	0.126 (3)
H21E	0.4967	0.0119	0.1359	0.036*	0.126 (3)
H21F	0.4164	−0.0783	0.1338	0.036*	0.126 (3)
C22B	0.5288 (9)	−0.1366 (12)	0.2266 (9)	0.0298 (18)*	0.126 (3)
H22E	0.5892	−0.1061	0.2424	0.036*	0.126 (3)
H22F	0.5288	−0.2068	0.1828	0.036*	0.126 (3)
C23B	0.4892 (9)	−0.1641 (9)	0.3233 (9)	0.0298 (18)*	0.126 (3)
H23E	0.4393	−0.2192	0.3098	0.036*	0.126 (3)
H23F	0.5333	−0.1975	0.3765	0.036*	0.126 (3)
C24B	0.4593 (9)	−0.0416 (11)	0.3526 (7)	0.0298 (18)*	0.126 (3)
H24E	0.4085	−0.0480	0.3899	0.036*	0.126 (3)
H24F	0.5071	−0.0007	0.3959	0.036*	0.126 (3)

### fac-[2-(2,2'-Bipyridin-6-yl)benzoic acid-κ2N,N']tricarbonylchloridorhenium(I) tetrahydrofuran monosolvate (II) Atomic displacement parameters (Å^2^)

**Table:**

	*U*^11^	*U*^22^	*U*^33^	*U*^12^	*U*^13^	*U*^23^
Re1	0.01365 (3)	0.01547 (3)	0.02146 (3)	−0.00104 (2)	0.00704 (2)	−0.00100 (2)
Cl1	0.02042 (15)	0.01814 (15)	0.02561 (17)	0.00341 (12)	0.00999 (12)	0.00509 (13)
O1	0.0344 (8)	0.0381 (9)	0.0991 (15)	−0.0196 (7)	0.0102 (9)	−0.0154 (9)
O2	0.0676 (11)	0.0336 (8)	0.0500 (9)	−0.0017 (7)	0.0453 (9)	0.0034 (7)
O3	0.0222 (6)	0.0359 (8)	0.0597 (10)	0.0097 (6)	0.0029 (6)	−0.0023 (7)
O4	0.0279 (6)	0.0249 (6)	0.0258 (6)	0.0039 (5)	0.0025 (5)	0.0017 (5)
O5	0.0250 (6)	0.0258 (6)	0.0395 (7)	0.0063 (5)	0.0105 (5)	−0.0027 (5)
N1	0.0201 (6)	0.0206 (6)	0.0159 (5)	0.0042 (5)	0.0022 (4)	−0.0032 (4)
N2	0.0152 (5)	0.0137 (5)	0.0142 (5)	0.0023 (4)	0.0046 (4)	0.0000 (4)
C1	0.0223 (8)	0.0249 (8)	0.0550 (12)	−0.0057 (7)	0.0106 (7)	−0.0059 (8)
C2	0.0330 (9)	0.0223 (7)	0.0385 (10)	0.0010 (6)	0.0234 (8)	0.0013 (7)
C3	0.0171 (7)	0.0261 (8)	0.0355 (9)	−0.0004 (6)	0.0062 (6)	−0.0043 (6)
C4	0.0297 (8)	0.0322 (9)	0.0258 (8)	0.0064 (7)	−0.0017 (6)	−0.0135 (7)
C5	0.0413 (11)	0.0496 (12)	0.0227 (8)	0.0188 (9)	−0.0034 (7)	−0.0165 (8)
C6	0.0434 (11)	0.0513 (12)	0.0160 (7)	0.0246 (10)	0.0085 (7)	0.0002 (7)
C7	0.0334 (9)	0.0310 (8)	0.0196 (7)	0.0122 (7)	0.0128 (6)	0.0070 (6)
C8	0.0215 (6)	0.0177 (6)	0.0151 (6)	0.0060 (5)	0.0061 (5)	0.0019 (5)
C9	0.0169 (6)	0.0148 (6)	0.0185 (6)	0.0019 (5)	0.0069 (5)	0.0013 (5)
C10	0.0230 (7)	0.0198 (7)	0.0370 (9)	−0.0019 (6)	0.0148 (6)	0.0016 (6)
C11	0.0215 (7)	0.0208 (7)	0.0525 (11)	−0.0063 (6)	0.0084 (7)	−0.0063 (7)
C12	0.0235 (7)	0.0229 (7)	0.0339 (9)	−0.0003 (6)	−0.0002 (6)	−0.0115 (6)
C13	0.0188 (6)	0.0177 (6)	0.0185 (6)	0.0043 (5)	0.0015 (5)	−0.0038 (5)
C14	0.0287 (8)	0.0259 (7)	0.0159 (6)	0.0078 (6)	0.0021 (6)	−0.0033 (6)
C15	0.0574 (13)	0.0442 (11)	0.0242 (9)	0.0312 (10)	0.0117 (8)	0.0077 (8)
C16	0.0722 (17)	0.0596 (15)	0.0237 (9)	0.0390 (14)	0.0119 (10)	0.0149 (9)
C17	0.0532 (13)	0.0509 (13)	0.0155 (7)	0.0185 (10)	0.0084 (8)	0.0037 (7)
C18	0.0319 (8)	0.0321 (9)	0.0182 (7)	0.0052 (7)	0.0037 (6)	−0.0051 (6)
C19	0.0226 (7)	0.0208 (7)	0.0176 (6)	0.0031 (5)	0.0004 (5)	−0.0046 (5)
C20	0.0148 (6)	0.0188 (6)	0.0273 (7)	−0.0012 (5)	0.0013 (5)	−0.0033 (6)
O6	0.0276 (13)	0.0409 (15)	0.061 (2)	0.0138 (11)	−0.0072 (19)	0.001 (2)
C21	0.0222 (14)	0.0421 (19)	0.058 (3)	0.0132 (13)	0.001 (2)	0.009 (2)
C22	0.0369 (19)	0.053 (2)	0.071 (3)	0.0202 (18)	0.010 (2)	0.022 (2)
C23	0.0303 (16)	0.080 (3)	0.049 (2)	0.0020 (18)	0.0129 (16)	0.028 (2)
C24	0.046 (2)	0.044 (2)	0.048 (2)	−0.0090 (17)	0.0000 (17)	0.0119 (17)
O6A	0.032 (3)	0.049 (3)	0.020 (2)	0.007 (2)	0.0013 (19)	0.008 (2)
C24A	0.032 (3)	0.049 (3)	0.020 (2)	0.007 (2)	0.0013 (19)	0.008 (2)
C23A	0.032 (3)	0.049 (3)	0.020 (2)	0.007 (2)	0.0013 (19)	0.008 (2)
C22A	0.032 (3)	0.049 (3)	0.020 (2)	0.007 (2)	0.0013 (19)	0.008 (2)
C21A	0.032 (3)	0.049 (3)	0.020 (2)	0.007 (2)	0.0013 (19)	0.008 (2)

### fac-[2-(2,2'-Bipyridin-6-yl)benzoic acid-κ2N,N']tricarbonylchloridorhenium(I) tetrahydrofuran monosolvate (II) Geometric parameters (Å, º)

**Table:**

Re1—Cl1	2.4875 (6)	C18—C19	1.399 (2)
Re1—N1	2.1580 (14)	C19—C20	1.492 (2)
Re1—N2	2.2008 (13)	O6—C21	1.458 (5)
Re1—C1	1.9074 (19)	O6—C24	1.469 (5)
Re1—C2	1.9244 (19)	C21—H21A	0.9900
Re1—C3	1.9146 (18)	C21—H21B	0.9900
O1—C1	1.150 (2)	C21—C22	1.508 (5)
O2—C2	1.151 (2)	C22—H22A	0.9900
O3—C3	1.149 (2)	C22—H22B	0.9900
O4—C20	1.206 (2)	C22—C23	1.558 (7)
O5—H5	0.90 (3)	C23—H23A	0.9900
O5—C20	1.333 (2)	C23—H23B	0.9900
N1—C4	1.347 (2)	C23—C24	1.507 (5)
N1—C8	1.347 (2)	C24—H24A	0.9900
N2—C9	1.3648 (18)	C24—H24B	0.9900
N2—C13	1.3506 (19)	O6A—C24A	1.441 (11)
C4—H4	0.9500	O6A—C21A	1.478 (13)
C4—C5	1.380 (3)	C24A—H24C	0.9900
C5—H5A	0.9500	C24A—H24D	0.9900
C5—C6	1.378 (4)	C24A—C23A	1.546 (12)
C6—H6	0.9500	C23A—H23C	0.9900
C6—C7	1.380 (3)	C23A—H23D	0.9900
C7—H7	0.9500	C23A—C22A	1.534 (13)
C7—C8	1.392 (2)	C22A—H22C	0.9900
C8—C9	1.473 (2)	C22A—H22D	0.9900
C9—C10	1.388 (2)	C22A—C21A	1.533 (13)
C10—H10	0.9500	C21A—H21C	0.9900
C10—C11	1.379 (3)	C21A—H21D	0.9900
C11—H11	0.9500	O6B—C21B	1.4432
C11—C12	1.367 (3)	O6B—C24B	1.4435
C12—H12	0.9500	C21B—H21E	0.9900
C12—C13	1.392 (2)	C21B—H21F	0.9900
C13—C14	1.493 (2)	C21B—C22B	1.5350
C14—C15	1.393 (2)	C22B—H22E	0.9900
C14—C19	1.400 (2)	C22B—H22F	0.9900
C15—H15	0.9500	C22B—C23B	1.5390
C15—C16	1.386 (3)	C23B—H23E	0.9900
C16—H16	0.9500	C23B—H23F	0.9900
C16—C17	1.389 (3)	C23B—C24B	1.5350
C17—H17	0.9500	C24B—H24E	0.9900
C17—C18	1.380 (3)	C24B—H24F	0.9900
C18—H18	0.9500		
			
N1—Re1—Cl1	82.45 (4)	C21—O6—C24	109.5 (3)
N1—Re1—N2	75.48 (5)	O6—C21—H21A	111.6
N2—Re1—Cl1	82.11 (4)	O6—C21—H21B	111.6
C1—Re1—Cl1	94.36 (6)	O6—C21—C22	101.1 (3)
C1—Re1—N1	94.91 (8)	H21A—C21—H21B	109.4
C1—Re1—N2	170.08 (7)	C22—C21—H21A	111.6
C1—Re1—C2	85.75 (9)	C22—C21—H21B	111.6
C1—Re1—C3	89.41 (8)	C21—C22—H22A	111.1
C2—Re1—Cl1	93.41 (6)	C21—C22—H22B	111.1
C2—Re1—N1	175.84 (7)	C21—C22—C23	103.5 (3)
C2—Re1—N2	103.67 (7)	H22A—C22—H22B	109.0
C3—Re1—Cl1	174.86 (5)	C23—C22—H22A	111.1
C3—Re1—N1	93.76 (7)	C23—C22—H22B	111.1
C3—Re1—N2	93.61 (6)	C22—C23—H23A	110.7
C3—Re1—C2	90.36 (8)	C22—C23—H23B	110.7
C20—O5—H5	109 (2)	H23A—C23—H23B	108.8
C4—N1—Re1	124.05 (13)	C24—C23—C22	105.4 (3)
C8—N1—Re1	117.26 (10)	C24—C23—H23A	110.7
C8—N1—C4	118.58 (15)	C24—C23—H23B	110.7
C9—N2—Re1	114.86 (10)	O6—C24—C23	104.5 (4)
C13—N2—Re1	127.46 (10)	O6—C24—H24A	110.8
C13—N2—C9	117.63 (13)	O6—C24—H24B	110.8
O1—C1—Re1	178.1 (2)	C23—C24—H24A	110.8
O2—C2—Re1	174.45 (19)	C23—C24—H24B	110.8
O3—C3—Re1	178.65 (18)	H24A—C24—H24B	108.9
N1—C4—H4	118.7	C24A—O6A—C21A	108.4 (10)
N1—C4—C5	122.6 (2)	O6A—C24A—H24C	110.3
C5—C4—H4	118.7	O6A—C24A—H24D	110.3
C4—C5—H5A	120.6	O6A—C24A—C23A	106.9 (9)
C6—C5—C4	118.87 (18)	H24C—C24A—H24D	108.6
C6—C5—H5A	120.6	C23A—C24A—H24C	110.3
C5—C6—H6	120.5	C23A—C24A—H24D	110.3
C5—C6—C7	119.08 (17)	C24A—C23A—H23C	111.6
C7—C6—H6	120.5	C24A—C23A—H23D	111.6
C6—C7—H7	120.3	H23C—C23A—H23D	109.4
C6—C7—C8	119.45 (19)	C22A—C23A—C24A	100.9 (8)
C8—C7—H7	120.3	C22A—C23A—H23C	111.6
N1—C8—C7	121.38 (15)	C22A—C23A—H23D	111.6
N1—C8—C9	115.86 (13)	C23A—C22A—H22C	111.5
C7—C8—C9	122.76 (15)	C23A—C22A—H22D	111.5
N2—C9—C8	116.40 (13)	H22C—C22A—H22D	109.3
N2—C9—C10	122.22 (14)	C21A—C22A—C23A	101.5 (9)
C10—C9—C8	121.36 (14)	C21A—C22A—H22C	111.5
C9—C10—H10	120.4	C21A—C22A—H22D	111.5
C11—C10—C9	119.24 (16)	O6A—C21A—C22A	99.9 (11)
C11—C10—H10	120.4	O6A—C21A—H21C	111.8
C10—C11—H11	120.5	O6A—C21A—H21D	111.8
C12—C11—C10	118.92 (16)	C22A—C21A—H21C	111.8
C12—C11—H11	120.5	C22A—C21A—H21D	111.8
C11—C12—H12	120.0	H21C—C21A—H21D	109.5
C11—C12—C13	120.07 (16)	C21B—O6B—C24B	109.5
C13—C12—H12	120.0	O6B—C21B—H21E	110.4
N2—C13—C12	121.85 (15)	O6B—C21B—H21F	110.4
N2—C13—C14	121.29 (14)	O6B—C21B—C22B	106.4
C12—C13—C14	116.49 (14)	H21E—C21B—H21F	108.6
C15—C14—C13	117.06 (15)	C22B—C21B—H21E	110.4
C15—C14—C19	118.97 (16)	C22B—C21B—H21F	110.4
C19—C14—C13	123.40 (15)	C21B—C22B—H22E	111.5
C14—C15—H15	119.5	C21B—C22B—H22F	111.5
C16—C15—C14	121.01 (18)	C21B—C22B—C23B	101.5
C16—C15—H15	119.5	H22E—C22B—H22F	109.3
C15—C16—H16	120.1	C23B—C22B—H22E	111.5
C15—C16—C17	119.87 (19)	C23B—C22B—H22F	111.5
C17—C16—H16	120.1	C22B—C23B—H23E	111.5
C16—C17—H17	120.1	C22B—C23B—H23F	111.5
C18—C17—C16	119.80 (18)	H23E—C23B—H23F	109.3
C18—C17—H17	120.1	C24B—C23B—C22B	101.5
C17—C18—H18	119.6	C24B—C23B—H23E	111.5
C17—C18—C19	120.72 (17)	C24B—C23B—H23F	111.5
C19—C18—H18	119.6	O6B—C24B—C23B	106.4
C14—C19—C20	120.28 (14)	O6B—C24B—H24E	110.4
C18—C19—C14	119.60 (15)	O6B—C24B—H24F	110.4
C18—C19—C20	120.12 (15)	C23B—C24B—H24E	110.4
O4—C20—O5	124.18 (16)	C23B—C24B—H24F	110.4
O4—C20—C19	124.17 (15)	H24E—C24B—H24F	108.6
O5—C20—C19	111.64 (14)		
			
Re1—N1—C4—C5	175.99 (14)	C13—N2—C9—C10	0.4 (2)
Re1—N1—C8—C7	−174.60 (12)	C13—C14—C15—C16	−173.3 (2)
Re1—N1—C8—C9	4.26 (17)	C13—C14—C19—C18	171.61 (16)
Re1—N2—C9—C8	−0.54 (16)	C13—C14—C19—C20	−8.9 (2)
Re1—N2—C9—C10	177.80 (12)	C14—C15—C16—C17	1.5 (4)
Re1—N2—C13—C12	−175.22 (11)	C14—C19—C20—O4	−8.5 (2)
Re1—N2—C13—C14	−2.4 (2)	C14—C19—C20—O5	171.69 (15)
N1—C4—C5—C6	−1.1 (3)	C15—C14—C19—C18	0.5 (3)
N1—C8—C9—N2	−2.41 (19)	C15—C14—C19—C20	179.93 (18)
N1—C8—C9—C10	179.23 (14)	C15—C16—C17—C18	−0.2 (4)
N2—C9—C10—C11	−2.4 (2)	C16—C17—C18—C19	−0.9 (4)
N2—C13—C14—C15	−88.2 (2)	C17—C18—C19—C14	0.8 (3)
N2—C13—C14—C19	100.55 (19)	C17—C18—C19—C20	−178.70 (18)
C4—N1—C8—C7	1.7 (2)	C18—C19—C20—O4	170.91 (16)
C4—N1—C8—C9	−179.42 (14)	C18—C19—C20—O5	−8.9 (2)
C4—C5—C6—C7	0.6 (3)	C19—C14—C15—C16	−1.6 (4)
C5—C6—C7—C8	1.0 (3)	O6—C21—C22—C23	−37.5 (4)
C6—C7—C8—N1	−2.2 (2)	C21—O6—C24—C23	−24.1 (5)
C6—C7—C8—C9	179.01 (15)	C21—C22—C23—C24	24.2 (4)
C7—C8—C9—N2	176.43 (14)	C22—C23—C24—O6	−1.0 (5)
C7—C8—C9—C10	−1.9 (2)	C24—O6—C21—C22	39.3 (5)
C8—N1—C4—C5	−0.1 (3)	O6A—C24A—C23A—C22A	−18.3 (16)
C8—C9—C10—C11	175.90 (15)	C24A—O6A—C21A—C22A	36.3 (19)
C9—N2—C13—C12	1.8 (2)	C24A—C23A—C22A—C21A	39.9 (14)
C9—N2—C13—C14	174.62 (13)	C23A—C22A—C21A—O6A	−46.9 (16)
C9—C10—C11—C12	2.1 (3)	C21A—O6A—C24A—C23A	−11.4 (19)
C10—C11—C12—C13	0.0 (3)	O6B—C21B—C22B—C23B	31.5
C11—C12—C13—N2	−2.1 (2)	C21B—O6B—C24B—C23B	−12.4
C11—C12—C13—C14	−175.19 (16)	C21B—C22B—C23B—C24B	−37.4
C12—C13—C14—C15	85.0 (2)	C22B—C23B—C24B—O6B	31.5
C12—C13—C14—C19	−86.3 (2)	C24B—O6B—C21B—C22B	−12.3
C13—N2—C9—C8	−177.94 (13)		

### fac-[2-(2,2'-Bipyridin-6-yl)benzoic acid-κ2N,N']tricarbonylchloridorhenium(I) tetrahydrofuran monosolvate (II) Hydrogen-bond geometry (Å, º)

**Table:**

*D*—H···*A*	*D*—H	H···*A*	*D*···*A*	*D*—H···*A*
O5—H5···O6	0.90 (3)	1.74 (4)	2.615 (4)	164 (3)
O5—H5···O6*A*	0.90 (3)	1.68 (4)	2.516 (15)	154 (3)
O5—H5···O6*B*	0.90 (3)	1.83 (4)	2.642 (9)	150 (3)
C5—H5*A*···Cl1^i^	0.95	2.80	3.371 (2)	120
C10—H10···Cl1^ii^	0.95	2.70	3.552 (2)	149
C12—H12···Cl1^iii^	0.95	2.76	3.524 (2)	138

Symmetry codes: (i) *x*, −*y*+3/2, *z*+1/2; (ii) −*x*, −*y*+1, −*z*+1; (iii) −*x*, *y*−1/2, −*z*+1/2.
